# WHY DO OLDER WOMEN LIMIT THEIR DRIVING MORE THAN OLDER MEN?

**DOI:** 10.1093/geroni/igad104.2684

**Published:** 2023-12-21

**Authors:** Anne Barrett, Hope Mimbs, Brianna Soulie, Jessica Noblitt, Cherish Michael

**Affiliations:** Florida State University, Tallahassee, Florida, United States; Florida State University, Tallahassee, Florida, United States; Florida State University, Tallahassee, Florida, United States; Florida State University, Tallahassee, Florida, United States; Florida State University, Tallahassee, Florida, United States

## Abstract

Older adults tend to avoid driving in situations like heavy traffic or bad weather, but women do so more than men. Explanations for this gender difference, however, have received limited research attention. Using data from an online survey of Floridians aged 50 and older that was conducted between December 2020 and April 2021 (n=3,726), we examined four potential explanations for women’s greater self-regulated driving: their worse health, their more frequent rides from others, their more negative attitudes about driving, and their less favorable assessments of their driving ability. The results of multivariate regression models predicting a scale of self-regulated driving revealed support for two of the explanations – rides from others and self-assessed driving ability. Women’s more frequent rides from family members accounted for approximately 10 percent of the association between gender and self-regulated driving, while women’s less favorable assessments of their driving ability accounted for nearly a third of it. In contrast, health and attitudes about driving did not operate as mediators. They did, however, predict self-regulated driving; having worse health or more negative driving attitudes was associated with more self-regulated driving. Our study identifies women’s less favorable assessments of their abilities as a primary determinant of their more rapid transition from driving. Older women’s transportation mobility could be better maintained through programs focused on enhancing their skills, as well as driving confidence.

